# *N*-(2,3-Dimethyl­phen­yl)-2-nitro­benzene­sulfonamide

**DOI:** 10.1107/S1600536812042845

**Published:** 2012-10-20

**Authors:** U. Chaithanya, Sabine Foro, B. Thimme Gowda

**Affiliations:** a Department of Chemistry, Mangalore University, Mangalagangotri 574 199, Mangalore, India; b Institute of Materials Science, Darmstadt University of Technology, Petersenstrasse 23, D-64287, Darmstadt, Germany

## Abstract

There are two independent mol­ecules in the asymmetric unit of the title compound, C_14_H_14_N_2_O_4_S. The N—H bonds are *syn* to the *ortho*-nitro groups in the sulfonyl benzene rings and *anti* to the methyl groups in the aniline benzene rings. The mol­ecules are twisted at the S—N bonds with torsion angles of −60.4 (3) and 58.8 (3)° in the two mol­ecules. The dihedral angles between the planes of the sulfonyl and the anilino benzene rings are 53.67 (8) and 56.99 (9)°. The amide H atoms of both mol­ecules are involved in an intra­molecular hydrogen bond, generating an *S*(7) motif. In the crystal, pairs of N—H⋯O(S) hydrogen bonds link like mol­ecules into inversion dimers.

## Related literature

For studies on the effects of substituents on the structures and other aspects of *N*-(ar­yl)-amides, see: Alkan *et al.* (2011 ▶); Bowes *et al.* (2003 ▶); Gowda *et al.* (1994 ▶); Saeed *et al.* (2010 ▶); Shahwar *et al.* (2012 ▶), of *N*-aryl­sulfonamides, see: Chaithanya *et al.* (2012 ▶); Gowda *et al.* (2002 ▶) and of *N*-chloro­aryl­sulfonamides, see: Gowda & Shetty (2004 ▶); Shetty & Gowda (2004 ▶). For hydrogen-bond motifs, see: Bernstein *et al.* (1995 ▶).
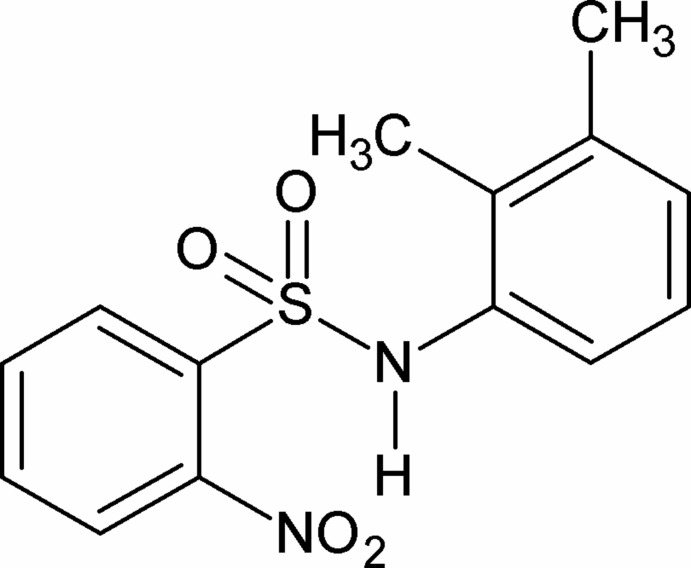


## Experimental

### 

#### Crystal data

C_14_H_14_N_2_O_4_S
*M* *_r_* = 306.33
Triclinic, 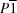

*a* = 8.0248 (9) Å
*b* = 12.633 (1) Å
*c* = 14.711 (1) Å
α = 88.205 (9)°
β = 80.818 (9)°
γ = 82.323 (9)°
*V* = 1459.0 (2) Å^3^
*Z* = 4
Mo *K*α radiation
μ = 0.24 mm^−1^
*T* = 293 K
0.44 × 0.40 × 0.24 mm

#### Data collection

Oxford Diffraction Xcalibur diffractometer with Sapphire CCD detector
Absorption correction: multi-scan (*CrysAlis RED*; Oxford Diffraction, 2009 ▶) *T*_min_ = 0.902, *T*_max_ = 0.945
10389 measured reflections
5936 independent reflections
3935 reflections with *I* > 2σ(*I*)
*R*_int_ = 0.018

#### Refinement

*R*[*F* ^2^ > 2σ(*F* ^2^)] = 0.062
*wR*(*F* ^2^) = 0.164
*S* = 1.02
5936 reflections
389 parameters
18 restraints
H atoms treated by a mixture of independent and constrained refinement
Δρ_max_ = 0.73 e Å^−3^
Δρ_min_ = −0.32 e Å^−3^

### 

Data collection: *CrysAlis CCD* (Oxford Diffraction, 2009 ▶); cell refinement: *CrysAlis CCD*; data reduction: *CrysAlis RED* (Oxford Diffraction, 2009 ▶); program(s) used to solve structure: *SHELXS97* (Sheldrick, 2008 ▶); program(s) used to refine structure: *SHELXL97* (Sheldrick, 2008 ▶); molecular graphics: *PLATON* (Spek, 2009 ▶); software used to prepare material for publication: *SHELXL97*.

## Supplementary Material

Crystal structure: contains datablock(s) I, global. DOI: 10.1107/S1600536812042845/sj5271sup1.cif

Structure factors: contains datablock(s) I. DOI: 10.1107/S1600536812042845/sj5271Isup2.hkl

Supplementary material file. DOI: 10.1107/S1600536812042845/sj5271Isup3.cml

Additional supplementary materials:  crystallographic information; 3D view; checkCIF report

## Figures and Tables

**Table 1:** Hydrogen-bond geometry (Å, °)

*D*—H⋯*A*	*D*—H	H⋯*A*	*D*⋯*A*	*D*—H⋯*A*
N1—H1*N*⋯O5^i^	0.83 (2)	2.36 (3)	3.056 (4)	142 (3)
N1—H1*N*⋯O7	0.83 (2)	2.45 (3)	3.011 (4)	126 (3)
N3—H3*N*⋯O3	0.85 (2)	2.40 (3)	3.012 (4)	129 (3)
N3—H3*N*⋯O1^ii^	0.85 (2)	2.41 (3)	3.070 (4)	135 (3)

Symmetry codes: (i) 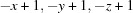; (ii) 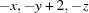.

**Fig. 1.**
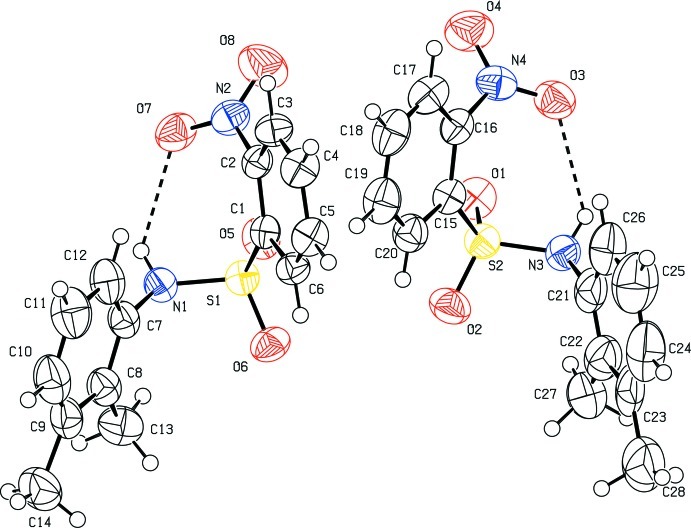
Molecular structure of the title compound, showing the atom labelling scheme and with displacement ellipsoids drawn at the 50% probability level and intramolecular hydrogen bonds drawn as dashed lines..

**Fig. 2.**
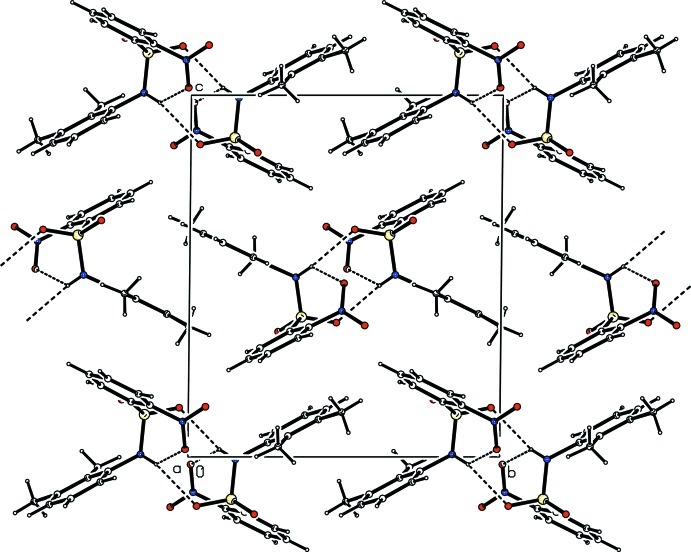
Crystal packing of the title compound with hydrogen bonds shown as dashed lines.

## supplementary crystallographic information

### Comment

As a part of studying the effect of substituents on the structures and other aspects of *N*-(aryl)-amides (Alkan *et al.*, 2011; Bowes *et al.*, 2003; Gowda *et al.*, 1994; Saeed *et al.*, 2010; Shahwar *et al.*, 2012), *N*-arylsulfonamides (Chaithanya *et al.*, 2012; Gowda *et al.*, 2002) and *N*-chloroarylsulfonamides (Gowda & Shetty, 2004; Shetty & Gowda, 2004), in the present work, the crystal structure of *N*-(2-methylphenyl)-2-nitrobenzenesulfonamide (I) has been determined (Fig. 1).

The asymmetric unit of the structure contains two independent molecules. The conformation of the N—H bonds are *syn* to the *ortho*-nitro groups in the sulfonyl benzene rings and *anti* to both the *ortho*- and *meta*-methyl groups in the anilino rings, compared to a *syn* conformation between the N—H bonds and the *ortho*-nitro groups in the sulfonyl benzene rings or the *ortho*-methyl group in the anilino ring observed in *N*-(2-methylphenyl)-2-nitrobenzenesulfonamide (II) (Chaithanya *et al.*, 2012). In (I) the molecules are twisted at the S—N bonds with the torsional angles of -60.37 (30) and 58.81 (34)°, compared to the value of 73.90 (26)° in (II). The dihedral angles between the sulfonyl and anilino rings are 53.67 (8) and 56.99 (9)°, compared to the value of 53.44 (14)° in (II).

The amide H-atoms each form intramolecular H-bonds with the O3 and O7 atoms of the *ortho*-nitro groups in the sulfonyl benzene rings, generating S(7) motifs (Bernstein *et al.*, 1995). Intermolecular H-bonds from amide H-atoms to sulfonyl oxygen atoms of a similar neighbouring molecule, generate R^2^_2_(8) inversion dimers (Table 1, Fig. 2.)

### Experimental

The title compound was prepared by treating 2-nitrobenzenesulfonylchloride with 2,3-dimethylaniline in the stoichiometric ratio and boiling the reaction mixture for 15 minutes. The reaction mixture was then cooled to room temperature and added to ice cold water (100 ml). The resultant solid *N*-(2,3-dimethylphenyl)-2-nitrobenzenesulfonamide was filtered under suction and washed thoroughly with cold water and dilute HCl to remove the excess sulfonylchloride and aniline, respectively. It was then recrystallized to constant melting point (138° C) from dilute ethanol. The purity of the compound was checked and characterized by its infrared spectrum.

Prism like light pink single crystals of the title compound used in X-ray diffraction studies were grown in ethanolic solution by slow evaporation of the solvent at room temperature.

### Refinement

H atoms bonded to C were positioned with idealized geometry using a riding model with the aromatic C—H = 0.93 Å, methyl C—H = 0.96 Å. The amino H atom was freely refined with the N—H distance restrained to 0.86 (2) Å. All H atoms were refined with isotropic displacement parameters set at 1.2 *U*_eq_(C-aromatic, N) and 1.5 *U*_eq_ (*C*-methyl)of the parent atom.

### Crystal data

**Table:**

C_14_H_14_N_2_O_4_S	*Z* = 4
*M**_r_* = 306.33	*F*(000) = 640
Triclinic, *P*1	*D*_x_ = 1.395 Mg m^−^^3^
Hall symbol: -P 1	Mo *K*α radiation, λ = 0.71073 Å
*a* = 8.0248 (9) Å	Cell parameters from 2511 reflections
*b* = 12.633 (1) Å	θ = 2.6–27.8°
*c* = 14.711 (1) Å	µ = 0.24 mm^−^^1^
α = 88.205 (9)°	*T* = 293 K
β = 80.818 (9)°	Prism, light pink
γ = 82.323 (9)°	0.44 × 0.40 × 0.24 mm
*V* = 1459.0 (2) Å^3^	

### Data collection

**Table:**

Oxford Diffraction Xcalibur diffractometer with Sapphire CCD detector	5936 independent reflections
Radiation source: fine-focus sealed tube	3935 reflections with *I* > 2σ(*I*)
Graphite monochromator	*R*_int_ = 0.018
Rotation method data acquisition using ω scans.	θ_max_ = 26.4°, θ_min_ = 2.6°
Absorption correction: multi-scan (*CrysAlis RED*; Oxford Diffraction, 2009)	*h* = −6→10
*T*_min_ = 0.902, *T*_max_ = 0.945	*k* = −15→15
10389 measured reflections	*l* = −18→18

### Refinement

**Table:**

Refinement on *F*^2^	Primary atom site location: structure-invariant direct methods
Least-squares matrix: full	Secondary atom site location: difference Fourier map
*R*[*F*^2^ > 2σ(*F*^2^)] = 0.062	Hydrogen site location: inferred from neighbouring sites
*wR*(*F*^2^) = 0.164	H atoms treated by a mixture of independent and constrained refinement
*S* = 1.02	*w* = 1/[σ^2^(*F*_o_^2^) + (0.0599*P*)^2^ + 1.4896*P*] where *P* = (*F*_o_^2^ + 2*F*_c_^2^)/3
5936 reflections	(Δ/σ)_max_ = 0.007
389 parameters	Δρ_max_ = 0.73 e Å^−^^3^
18 restraints	Δρ_min_ = −0.32 e Å^−^^3^

### Special details

**Table:**

Geometry. All e.s.d.'s (except the e.s.d. in the dihedral angle between two l.s. planes) are estimated using the full covariance matrix. The cell e.s.d.'s are taken into account individually in the estimation of e.s.d.'s in distances, angles and torsion angles; correlations between e.s.d.'s in cell parameters are only used when they are defined by crystal symmetry. An approximate (isotropic) treatment of cell e.s.d.'s is used for estimating e.s.d.'s involving l.s. planes.
Refinement. Refinement of *F*^2^ against ALL reflections. The weighted *R*-factor *wR* and goodness of fit *S* are based on *F*^2^, conventional *R*-factors *R* are based on *F*, with *F* set to zero for negative *F*^2^. The threshold expression of *F*^2^ > σ(*F*^2^) is used only for calculating *R*-factors(gt) *etc*. and is not relevant to the choice of reflections for refinement. *R*-factors based on *F*^2^ are statistically about twice as large as those based on *F*, and *R*- factors based on ALL data will be even larger.

### Fractional atomic coordinates and isotropic or equivalent isotropic displacement parameters (Å^2^)

**Table:**

	*x*	*y*	*z*	*U*_iso_*/*U*_eq_	
S1	0.59078 (10)	0.35977 (6)	0.38703 (6)	0.0426 (2)	
O5	0.5488 (3)	0.47186 (18)	0.37132 (17)	0.0537 (6)	
O6	0.5067 (3)	0.2854 (2)	0.34621 (17)	0.0561 (6)	
O7	0.8170 (3)	0.4954 (2)	0.47995 (19)	0.0637 (7)	
O8	0.9658 (5)	0.5631 (2)	0.3634 (2)	0.0896 (10)	
N1	0.5588 (4)	0.3406 (2)	0.49664 (19)	0.0454 (7)	
H1N	0.581 (5)	0.391 (2)	0.526 (2)	0.055*	
N2	0.9020 (4)	0.4890 (2)	0.4036 (2)	0.0523 (7)	
C1	0.8118 (4)	0.3235 (2)	0.3470 (2)	0.0372 (7)	
C2	0.9393 (4)	0.3844 (2)	0.3586 (2)	0.0399 (7)	
C3	1.1079 (4)	0.3496 (3)	0.3287 (2)	0.0488 (8)	
H3	1.1912	0.3911	0.3378	0.059*	
C4	1.1527 (4)	0.2528 (3)	0.2851 (3)	0.0533 (9)	
H4	1.2668	0.2281	0.2654	0.064*	
C5	1.0292 (4)	0.1927 (3)	0.2706 (2)	0.0503 (9)	
H5	1.0599	0.1279	0.2402	0.060*	
C6	0.8602 (4)	0.2276 (3)	0.3009 (2)	0.0437 (8)	
H6	0.7776	0.1864	0.2904	0.052*	
C7	0.5971 (5)	0.2352 (3)	0.5374 (2)	0.0476 (8)	
C8	0.4693 (5)	0.1718 (3)	0.5629 (2)	0.0523 (9)	
C9	0.5109 (6)	0.0721 (3)	0.6048 (2)	0.0582 (10)	
C10	0.6782 (7)	0.0421 (3)	0.6172 (3)	0.0748 (13)	
H10	0.7065	−0.0243	0.6438	0.090*	
C11	0.8033 (6)	0.1053 (4)	0.5925 (3)	0.0781 (13)	
H11	0.9138	0.0822	0.6028	0.094*	
C12	0.7653 (5)	0.2033 (3)	0.5522 (3)	0.0620 (10)	
H12	0.8491	0.2475	0.5351	0.074*	
C13	0.2916 (5)	0.2082 (3)	0.5485 (3)	0.0708 (12)	
H13A	0.2840	0.2797	0.5239	0.106*	
H13B	0.2167	0.2069	0.6063	0.106*	
H13C	0.2591	0.1615	0.5061	0.106*	
C14	0.3784 (7)	0.0013 (3)	0.6390 (3)	0.0865 (15)	
H14A	0.2969	0.0369	0.6869	0.130*	
H14B	0.4316	−0.0640	0.6627	0.130*	
H14C	0.3218	−0.0142	0.5892	0.130*	
S2	0.02193 (11)	0.85930 (7)	0.11713 (6)	0.0481 (2)	
O1	−0.0211 (3)	0.97056 (19)	0.13593 (18)	0.0594 (7)	
O2	−0.0880 (3)	0.7857 (2)	0.15857 (19)	0.0648 (7)	
O3	0.3080 (3)	0.9908 (2)	0.0211 (2)	0.0657 (7)	
O4	0.3890 (4)	1.0547 (2)	0.1388 (2)	0.0822 (9)	
N3	0.0419 (4)	0.8477 (2)	0.0069 (2)	0.0509 (7)	
H3N	0.091 (4)	0.897 (2)	−0.022 (2)	0.061*	
N4	0.3487 (4)	0.9821 (2)	0.0977 (2)	0.0534 (8)	
C15	0.2213 (4)	0.8163 (3)	0.1527 (2)	0.0434 (8)	
C16	0.3568 (4)	0.8767 (3)	0.1430 (2)	0.0447 (8)	
C17	0.5065 (5)	0.8400 (3)	0.1742 (3)	0.0572 (10)	
H17	0.5952	0.8815	0.1671	0.069*	
C18	0.5242 (5)	0.7411 (3)	0.2163 (3)	0.0650 (11)	
H18	0.6259	0.7152	0.2368	0.078*	
C19	0.3936 (6)	0.6811 (3)	0.2282 (3)	0.0643 (11)	
H19	0.4065	0.6146	0.2570	0.077*	
C20	0.2406 (5)	0.7184 (3)	0.1975 (2)	0.0543 (9)	
H20	0.1511	0.6774	0.2071	0.065*	
C21	0.0992 (6)	0.7447 (3)	−0.0398 (3)	0.0652 (8)	
C22	−0.0109 (7)	0.6796 (3)	−0.0580 (3)	0.0756 (10)	
C23	0.0528 (8)	0.5828 (3)	−0.1033 (3)	0.0824 (11)	
C24	0.2297 (9)	0.5572 (4)	−0.1317 (3)	0.1028 (14)	
H24	0.2724	0.4932	−0.1617	0.123*	
C25	0.3376 (9)	0.6261 (5)	−0.1152 (4)	0.1085 (16)	
H25	0.4535	0.6084	−0.1358	0.130*	
C26	0.2839 (7)	0.7197 (4)	−0.0697 (3)	0.0804 (10)	
H26	0.3598	0.7654	−0.0583	0.096*	
C27	−0.1925 (7)	0.7109 (4)	−0.0284 (3)	0.0878 (12)	
H27A	−0.2364	0.6588	0.0146	0.132*	
H27B	−0.2505	0.7151	−0.0809	0.132*	
H27C	−0.2102	0.7794	0.0006	0.132*	
C28	−0.0634 (10)	0.5087 (4)	−0.1236 (4)	0.124 (2)	
H28A	−0.1409	0.5443	−0.1616	0.186*	
H28B	−0.1265	0.4858	−0.0670	0.186*	
H28C	0.0015	0.4477	−0.1554	0.186*	

### Atomic displacement parameters (Å^2^)

**Table:**

	*U*^11^	*U*^22^	*U*^33^	*U*^12^	*U*^13^	*U*^23^
S1	0.0351 (4)	0.0445 (5)	0.0468 (5)	0.0025 (3)	−0.0089 (3)	0.0013 (4)
O5	0.0494 (14)	0.0469 (14)	0.0596 (16)	0.0113 (11)	−0.0094 (11)	0.0082 (11)
O6	0.0397 (13)	0.0690 (16)	0.0628 (16)	−0.0087 (12)	−0.0150 (11)	−0.0083 (13)
O7	0.0597 (16)	0.0675 (18)	0.0631 (18)	−0.0091 (13)	−0.0023 (14)	−0.0225 (14)
O8	0.121 (3)	0.0455 (16)	0.096 (2)	−0.0205 (17)	0.009 (2)	−0.0026 (16)
N1	0.0487 (16)	0.0413 (16)	0.0442 (17)	−0.0036 (13)	−0.0029 (13)	−0.0016 (12)
N2	0.0528 (18)	0.0457 (18)	0.059 (2)	−0.0038 (14)	−0.0128 (16)	−0.0060 (15)
C1	0.0358 (16)	0.0378 (17)	0.0368 (17)	0.0033 (13)	−0.0091 (13)	0.0014 (13)
C2	0.0436 (18)	0.0376 (17)	0.0385 (18)	−0.0004 (14)	−0.0108 (14)	−0.0010 (13)
C3	0.0368 (18)	0.054 (2)	0.058 (2)	−0.0060 (15)	−0.0127 (16)	−0.0012 (17)
C4	0.0373 (19)	0.059 (2)	0.060 (2)	0.0084 (16)	−0.0090 (16)	−0.0070 (18)
C5	0.050 (2)	0.044 (2)	0.054 (2)	0.0068 (16)	−0.0076 (17)	−0.0082 (16)
C6	0.0427 (18)	0.0430 (19)	0.0464 (19)	−0.0058 (15)	−0.0099 (15)	−0.0005 (15)
C7	0.060 (2)	0.0387 (18)	0.0400 (19)	−0.0022 (16)	0.0001 (16)	−0.0016 (14)
C8	0.058 (2)	0.048 (2)	0.046 (2)	−0.0024 (17)	0.0030 (17)	−0.0060 (16)
C9	0.084 (3)	0.045 (2)	0.042 (2)	−0.005 (2)	−0.0016 (19)	−0.0009 (16)
C10	0.102 (4)	0.060 (3)	0.056 (3)	0.001 (3)	−0.003 (2)	0.006 (2)
C11	0.072 (3)	0.086 (3)	0.070 (3)	0.012 (3)	−0.015 (2)	0.013 (2)
C12	0.066 (3)	0.064 (3)	0.051 (2)	0.005 (2)	−0.0094 (19)	0.0092 (19)
C13	0.062 (3)	0.067 (3)	0.082 (3)	−0.011 (2)	−0.006 (2)	−0.003 (2)
C14	0.123 (4)	0.060 (3)	0.075 (3)	−0.030 (3)	0.001 (3)	0.004 (2)
S2	0.0398 (5)	0.0510 (5)	0.0532 (5)	−0.0027 (4)	−0.0099 (4)	0.0027 (4)
O1	0.0505 (15)	0.0545 (15)	0.0684 (17)	0.0094 (12)	−0.0072 (12)	−0.0072 (13)
O2	0.0489 (15)	0.0765 (18)	0.0721 (18)	−0.0202 (13)	−0.0129 (13)	0.0186 (14)
O3	0.0609 (17)	0.0663 (18)	0.0726 (19)	−0.0141 (13)	−0.0165 (15)	0.0145 (14)
O4	0.096 (2)	0.0583 (18)	0.093 (2)	−0.0181 (16)	−0.0072 (18)	−0.0174 (16)
N3	0.0561 (19)	0.0487 (15)	0.0506 (18)	−0.0072 (13)	−0.0181 (15)	0.0080 (12)
N4	0.0405 (17)	0.0504 (19)	0.066 (2)	−0.0044 (14)	0.0013 (15)	−0.0054 (16)
C15	0.0411 (18)	0.0443 (19)	0.0436 (19)	0.0023 (15)	−0.0087 (14)	−0.0044 (15)
C16	0.0402 (18)	0.0479 (19)	0.0440 (19)	0.0029 (15)	−0.0058 (14)	−0.0096 (15)
C17	0.041 (2)	0.065 (2)	0.064 (2)	0.0031 (17)	−0.0093 (17)	−0.017 (2)
C18	0.056 (2)	0.077 (3)	0.059 (2)	0.014 (2)	−0.021 (2)	−0.009 (2)
C19	0.075 (3)	0.056 (2)	0.060 (3)	0.012 (2)	−0.022 (2)	0.0044 (19)
C20	0.060 (2)	0.052 (2)	0.051 (2)	−0.0042 (18)	−0.0131 (18)	0.0023 (17)
C21	0.0985 (19)	0.0527 (17)	0.047 (2)	−0.0022 (15)	−0.0262 (19)	0.0070 (15)
C22	0.1152 (19)	0.056 (2)	0.060 (3)	−0.0115 (17)	−0.027 (2)	0.0067 (16)
C23	0.160 (3)	0.0523 (19)	0.038 (2)	−0.012 (2)	−0.030 (2)	0.0094 (15)
C24	0.167 (4)	0.073 (3)	0.061 (3)	0.011 (2)	−0.017 (3)	−0.002 (2)
C25	0.130 (3)	0.100 (4)	0.084 (4)	0.018 (2)	−0.008 (3)	−0.019 (3)
C26	0.0994 (19)	0.082 (3)	0.056 (3)	0.011 (2)	−0.019 (2)	0.000 (2)
C27	0.1075 (19)	0.092 (3)	0.068 (3)	−0.023 (2)	−0.019 (2)	0.019 (2)
C28	0.210 (7)	0.094 (4)	0.077 (4)	−0.045 (4)	−0.030 (4)	0.016 (3)

### Geometric parameters (Å, º)

**Table:**

S1—O6	1.426 (2)	S2—O2	1.424 (3)
S1—O5	1.431 (2)	S2—O1	1.427 (2)
S1—N1	1.608 (3)	S2—N3	1.613 (3)
S1—C1	1.780 (3)	S2—C15	1.774 (3)
O7—N2	1.216 (4)	O3—N4	1.220 (4)
O8—N2	1.218 (4)	O4—N4	1.219 (4)
N1—C7	1.458 (4)	N3—C21	1.474 (5)
N1—H1N	0.829 (18)	N3—H3N	0.846 (18)
N2—C2	1.470 (4)	N4—C16	1.469 (4)
C1—C6	1.385 (4)	C15—C20	1.385 (5)
C1—C2	1.394 (4)	C15—C16	1.396 (5)
C2—C3	1.371 (4)	C16—C17	1.372 (5)
C3—C4	1.377 (5)	C17—C18	1.377 (5)
C3—H3	0.9300	C17—H17	0.9300
C4—C5	1.373 (5)	C18—C19	1.360 (6)
C4—H4	0.9300	C18—H18	0.9300
C5—C6	1.375 (5)	C19—C20	1.394 (5)
C5—H5	0.9300	C19—H19	0.9300
C6—H6	0.9300	C20—H20	0.9300
C7—C8	1.382 (5)	C21—C22	1.346 (6)
C7—C12	1.405 (5)	C21—C26	1.472 (7)
C8—C9	1.409 (5)	C22—C23	1.406 (6)
C8—C13	1.485 (5)	C22—C27	1.457 (7)
C9—C10	1.384 (6)	C23—C24	1.411 (8)
C9—C14	1.495 (6)	C23—C28	1.475 (7)
C10—C11	1.363 (6)	C24—C25	1.360 (8)
C10—H10	0.9300	C24—H24	0.9300
C11—C12	1.374 (5)	C25—C26	1.361 (6)
C11—H11	0.9300	C25—H25	0.9300
C12—H12	0.9300	C26—H26	0.9300
C13—H13A	0.9600	C27—H27A	0.9600
C13—H13B	0.9600	C27—H27B	0.9600
C13—H13C	0.9600	C27—H27C	0.9600
C14—H14A	0.9600	C28—H28A	0.9600
C14—H14B	0.9600	C28—H28B	0.9600
C14—H14C	0.9600	C28—H28C	0.9600
			
O6—S1—O5	119.75 (15)	O2—S2—O1	119.82 (16)
O6—S1—N1	107.92 (16)	O2—S2—N3	107.96 (17)
O5—S1—N1	106.93 (14)	O1—S2—N3	106.38 (16)
O6—S1—C1	105.38 (14)	O2—S2—C15	105.30 (16)
O5—S1—C1	108.58 (15)	O1—S2—C15	108.09 (15)
N1—S1—C1	107.78 (15)	N3—S2—C15	108.98 (16)
C7—N1—S1	121.6 (2)	C21—N3—S2	122.4 (2)
C7—N1—H1N	114 (3)	C21—N3—H3N	111 (3)
S1—N1—H1N	113 (3)	S2—N3—H3N	112 (3)
O7—N2—O8	124.1 (3)	O4—N4—O3	124.5 (3)
O7—N2—C2	118.5 (3)	O4—N4—C16	116.9 (3)
O8—N2—C2	117.3 (3)	O3—N4—C16	118.5 (3)
C6—C1—C2	117.7 (3)	C20—C15—C16	117.8 (3)
C6—C1—S1	117.5 (2)	C20—C15—S2	117.6 (3)
C2—C1—S1	124.8 (2)	C16—C15—S2	124.5 (3)
C3—C2—C1	121.6 (3)	C17—C16—C15	121.7 (3)
C3—C2—N2	116.0 (3)	C17—C16—N4	116.2 (3)
C1—C2—N2	122.4 (3)	C15—C16—N4	122.1 (3)
C2—C3—C4	119.4 (3)	C16—C17—C18	119.4 (4)
C2—C3—H3	120.3	C16—C17—H17	120.3
C4—C3—H3	120.3	C18—C17—H17	120.3
C5—C4—C3	120.1 (3)	C19—C18—C17	120.3 (4)
C5—C4—H4	120.0	C19—C18—H18	119.8
C3—C4—H4	120.0	C17—C18—H18	119.8
C4—C5—C6	120.4 (3)	C18—C19—C20	120.6 (4)
C4—C5—H5	119.8	C18—C19—H19	119.7
C6—C5—H5	119.8	C20—C19—H19	119.7
C5—C6—C1	120.8 (3)	C15—C20—C19	120.2 (4)
C5—C6—H6	119.6	C15—C20—H20	119.9
C1—C6—H6	119.6	C19—C20—H20	119.9
C8—C7—C12	122.1 (3)	C22—C21—C26	121.8 (4)
C8—C7—N1	120.2 (3)	C22—C21—N3	122.2 (4)
C12—C7—N1	117.6 (3)	C26—C21—N3	115.9 (4)
C7—C8—C9	118.3 (4)	C21—C22—C23	119.1 (5)
C7—C8—C13	121.3 (3)	C21—C22—C27	118.8 (4)
C9—C8—C13	120.4 (4)	C23—C22—C27	122.0 (5)
C10—C9—C8	118.2 (4)	C22—C23—C24	119.7 (5)
C10—C9—C14	120.1 (4)	C22—C23—C28	120.8 (6)
C8—C9—C14	121.6 (4)	C24—C23—C28	119.4 (5)
C11—C10—C9	123.2 (4)	C25—C24—C23	119.9 (5)
C11—C10—H10	118.4	C25—C24—H24	120.0
C9—C10—H10	118.4	C23—C24—H24	120.0
C10—C11—C12	119.6 (4)	C24—C25—C26	123.0 (6)
C10—C11—H11	120.2	C24—C25—H25	118.5
C12—C11—H11	120.2	C26—C25—H25	118.5
C11—C12—C7	118.6 (4)	C25—C26—C21	116.3 (5)
C11—C12—H12	120.7	C25—C26—H26	121.8
C7—C12—H12	120.7	C21—C26—H26	121.8
C8—C13—H13A	109.5	C22—C27—H27A	109.5
C8—C13—H13B	109.5	C22—C27—H27B	109.5
H13A—C13—H13B	109.5	H27A—C27—H27B	109.5
C8—C13—H13C	109.5	C22—C27—H27C	109.5
H13A—C13—H13C	109.5	H27A—C27—H27C	109.5
H13B—C13—H13C	109.5	H27B—C27—H27C	109.5
C9—C14—H14A	109.5	C23—C28—H28A	109.5
C9—C14—H14B	109.5	C23—C28—H28B	109.5
H14A—C14—H14B	109.5	H28A—C28—H28B	109.5
C9—C14—H14C	109.5	C23—C28—H28C	109.5
H14A—C14—H14C	109.5	H28A—C28—H28C	109.5
H14B—C14—H14C	109.5	H28B—C28—H28C	109.5
			
O6—S1—N1—C7	53.0 (3)	O2—S2—N3—C21	−55.1 (3)
O5—S1—N1—C7	−177.0 (3)	O1—S2—N3—C21	175.1 (3)
C1—S1—N1—C7	−60.4 (3)	C15—S2—N3—C21	58.8 (3)
O6—S1—C1—C6	−10.1 (3)	O2—S2—C15—C20	9.5 (3)
O5—S1—C1—C6	−139.6 (2)	O1—S2—C15—C20	138.7 (3)
N1—S1—C1—C6	104.9 (3)	N3—S2—C15—C20	−106.1 (3)
O6—S1—C1—C2	169.9 (3)	O2—S2—C15—C16	−166.9 (3)
O5—S1—C1—C2	40.5 (3)	O1—S2—C15—C16	−37.7 (3)
N1—S1—C1—C2	−75.0 (3)	N3—S2—C15—C16	77.5 (3)
C6—C1—C2—C3	−2.3 (5)	C20—C15—C16—C17	1.8 (5)
S1—C1—C2—C3	177.7 (3)	S2—C15—C16—C17	178.2 (3)
C6—C1—C2—N2	177.9 (3)	C20—C15—C16—N4	−179.2 (3)
S1—C1—C2—N2	−2.1 (4)	S2—C15—C16—N4	−2.8 (5)
O7—N2—C2—C3	−126.1 (3)	O4—N4—C16—C17	−50.8 (4)
O8—N2—C2—C3	50.4 (4)	O3—N4—C16—C17	126.9 (3)
O7—N2—C2—C1	53.8 (4)	O4—N4—C16—C15	130.2 (3)
O8—N2—C2—C1	−129.7 (4)	O3—N4—C16—C15	−52.2 (4)
C1—C2—C3—C4	0.7 (5)	C15—C16—C17—C18	0.0 (5)
N2—C2—C3—C4	−179.4 (3)	N4—C16—C17—C18	−179.1 (3)
C2—C3—C4—C5	1.0 (5)	C16—C17—C18—C19	−1.1 (6)
C3—C4—C5—C6	−1.1 (6)	C17—C18—C19—C20	0.4 (6)
C4—C5—C6—C1	−0.4 (5)	C16—C15—C20—C19	−2.4 (5)
C2—C1—C6—C5	2.1 (5)	S2—C15—C20—C19	−179.1 (3)
S1—C1—C6—C5	−177.9 (3)	C18—C19—C20—C15	1.4 (6)
S1—N1—C7—C8	−97.5 (3)	S2—N3—C21—C22	92.1 (4)
S1—N1—C7—C12	84.6 (4)	S2—N3—C21—C26	−90.9 (4)
C12—C7—C8—C9	−0.1 (5)	C26—C21—C22—C23	2.8 (6)
N1—C7—C8—C9	−177.9 (3)	N3—C21—C22—C23	179.6 (3)
C12—C7—C8—C13	178.6 (3)	C26—C21—C22—C27	−178.3 (4)
N1—C7—C8—C13	0.8 (5)	N3—C21—C22—C27	−1.6 (6)
C7—C8—C9—C10	−0.9 (5)	C21—C22—C23—C24	−2.1 (6)
C13—C8—C9—C10	−179.6 (4)	C27—C22—C23—C24	179.1 (4)
C7—C8—C9—C14	176.7 (3)	C21—C22—C23—C28	179.3 (4)
C13—C8—C9—C14	−2.0 (6)	C27—C22—C23—C28	0.5 (6)
C8—C9—C10—C11	1.3 (6)	C22—C23—C24—C25	0.0 (7)
C14—C9—C10—C11	−176.3 (4)	C28—C23—C24—C25	178.6 (5)
C9—C10—C11—C12	−0.8 (7)	C23—C24—C25—C26	1.6 (8)
C10—C11—C12—C7	−0.2 (6)	C24—C25—C26—C21	−0.9 (8)
C8—C7—C12—C11	0.6 (6)	C22—C21—C26—C25	−1.3 (6)
N1—C7—C12—C11	178.5 (3)	N3—C21—C26—C25	−178.3 (4)

### Hydrogen-bond geometry (Å, º)

**Table:**

*D*—H···*A*	*D*—H	H···*A*	*D*···*A*	*D*—H···*A*
N1—H1*N*···O5^i^	0.83 (2)	2.36 (3)	3.056 (4)	142 (3)
N1—H1*N*···O7	0.83 (2)	2.45 (3)	3.011 (4)	126 (3)
N3—H3*N*···O3	0.85 (2)	2.40 (3)	3.012 (4)	129 (3)
N3—H3*N*···O1^ii^	0.85 (2)	2.41 (3)	3.070 (4)	135 (3)

Symmetry codes: (i) −*x*+1, −*y*+1, −*z*+1; (ii) −*x*, −*y*+2, −*z*.
